# *Anncaliia algerae* Microsporidial Myositis

**DOI:** 10.3201/eid2002.131126

**Published:** 2014-02

**Authors:** Matthew R. Watts, Renee C.F. Chan, Elaine Y.L. Cheong, Susan Brammah, Kate R. Clezy, Chiwai Tong, Deborah Marriott, Cameron E. Webb, Bobby Chacko, Vivienne Tobias, Alexander C. Outhred, Andrew S. Field, Michael V. Prowse, James V. Bertouch, Damien Stark, Stephen W. Reddel

**Affiliations:** Centre for Infectious Diseases and Microbiology Public Health, Westmead, New South Wales, Australia (M.R. Watts, C.E. Webb, A.C. Outhred);; Pathology West–Institute for Clinical Pathology and Medical Research Westmead, Westmead (M.R. Watts, C.E. Webb);; University of Sydney, Sydney, New South Wales, Australia (M.R. Watts, C.E. Webb, E.Y.L. Cheong, A.C. Outhred, S.W. Reddel);; Concord Repatriation General Hospital, Concord West, New South Wales, Australia (R.C.F. Chan, E.Y.L. Cheong, S. Brammah, S.W. Reddel);; Prince of Wales Hospital, Randwick, New South Wales, Australia (K.R. Clezy, C. Tong, J.V. Bertouch);; St Vincent’s Hospital, Darlinghurst, New South Wales, Australia (D. Marriott, A.S. Field, D. Stark);; John Hunter Hospital, Newcastle, New South Wales, Australia (B. Chacko);; Liverpool Hospital, Liverpool, New South Wales, Australia (V. Tobias);; Port Macquarie Base Hospital, Port Macquarie, New South Wales, Australia (M.V. Prowse)

**Keywords:** microporidia, insects, myositis, infection, arthritis rheumatoid, solid-organ transplantation, Anncaliia algerae, Australia

## Abstract

The insect microsporidian *Anncaliia algerae* was first described in 2004 as a cause of fatal myositis in an immunosuppressed person from Pennsylvania, USA. Two cases were subsequently reported, and we detail 2 additional cases, including the only nonfatal case. We reviewed all 5 case histories with respect to clinical characteristics, diagnosis, and management and summarized organism life cycle and epidemiology. Before infection, all case-patients were using immunosuppressive medications for rheumatoid arthritis or solid-organ transplantation. Four of the 5 case-patients were from Australia. All diagnoses were confirmed by skeletal muscle biopsy; however, peripheral nerves and other tissues may be infected. The surviving patient received albendazole and had a reduction of immunosuppressive medications and measures to prevent complications. Although insects are the natural hosts for *A. algerae*, human contact with water contaminated by spores may be a mode of transmission. *A. algerae* has emerged as a cause of myositis, particularly in coastal Australia.

## Abstract 2

Immunosuppression is a risk factor for serious infection in humans.

Microsporidia is a phylum of eukaryotes that contains almost 160 genera (*1*). Related to fungi, they are obligate intracellular organisms that spread among hosts through a spore stage (*1*). The microsporidian *Anncaliia algerae*, former genera *Nosema* and *Brachiola*, is an emerging human pathogen that primarily infects insects (*2*–*7*). *A. algerae* has caused severe myositis in patients taking immunosuppressive medication for rheumatoid arthritis or solid-organ transplantation (*3*,*5*,*8*). It also has led to skin abscesses and an infection of the false vocal cord in patients receiving chemotherapy for hematologic malignancies and caused keratitis in a man with no significant medical history (*2*,*4*,*6*).

Two other species of *Anncaliia* are reported to cause myositis in humans (*9*). *A. vesicularum* caused infection localized to the skeletal muscle in a patient with HIV (*9*). Another *Anncaliia* species, probably *A. connori*, led to disseminated infection in an infant who had thymic dysplasia and malabsorption syndrome (*9*,*10*). Autopsy demonstrated microsporidia in myocytes from the heart and diaphragm and the muscularis of the gut and arteries (*10*). Organisms also were seen in the alveolar septae, renal tubular cells, and parenchyma of the adrenal glands and liver (*10*). Microsporidia of the genera, *Trachipleistophora*, *Pleistophora*, and *Tubulinosema* also can cause myositis in immunocompromised hosts (*11*–*17*); the infection can be localized or part of a disseminated infection. A recent case report from Thailand described an infection caused by a novel microsporidia, related to *Endoreticulatus* spp, in the skeletal muscle, urinary tract, and bone marrow of a previously healthy man (*18*).

*A. algerae* myositis was first described in 2004 in a patient from Pennsylvania, USA, who had rheumatoid arthritis (*3*). Two patients in subsequent reports had histories of lung transplantation, 1 with a recent renal transplantation (*5*,*8*). We describe 2 additional cases of *A. algerae* myositis in patients with histories of rheumatoid arthritis, including 1 who survived. Four of these 5 case-patients were from the east coast of Australia. We have reviewed all 5 case histories with respect to clinical characteristics, diagnosis, and management (Table 1,2) and summarized organism life cycle and epidemiology.

**Table 1:** Epidemiologic and clinical characteristics of *Anncaliia algerae* myositis cases-patients*

Characteristic	Case-patient A	Case-patient B	Case-patient 1 (*3*)	Case-patient 2 (*5*)	Case-patient 3 (*8*)
Age, y/sex	67/M	66/M	57/F	49/M	56/M
Residence	Port Macquarie, NSW, Australia	Sydney, NSW, Australia	PA, USA	Woolongong, NSW, Australia	Rutherford, NSW, Australia
Distance from residence to open land, m	<100 to golf course	<100 to golf course	ND	<200 to coastal woodland	<100 to golf course
Background illness	RA	RA	RA, T2DM	Lung Tx, T1DM, CD	Lung Tx, kidney Tx
Immunosuppression	MTX, CS, LEF, ETN	MTX	MTX, CS, LEF, IFX	AZ, TAC, MMF, CS	TAC, MMF, CS
Fever	Yes	Yes	Yes	Yes	Yes
Fatigue	Yes	Yes	Yes	Yes	Yes
Weight loss	Yes	Yes	ND	Yes	Yes
Weakness	Yes	Yes	Yes	Yes	Yes
Generalized pain	Yes	Yes	Yes	Yes	Yes
Dysphagia	Yes	Yes	ND	Yes	Yes
Glossitis	Yes	Yes	ND	Yes	Yes
Peripheral edema	Yes	Yes	ND	Yes	Yes
Diarrhea	Yes	No	ND	Yes	Yes
CNS abnormalities	No	Delirium	Cerebrovascular infarction	Delirium, seizures†	Delirium

*NSW, New South Wales; PA, Pennsylvania; ND, not described in publication; RA, rheumatoid arthritis; T2DM, type 2 diabetes mellitus. Tx, transplantation; T1DM, type 1 diabetes mellitus; CD, Crohn disease; MTX, methotrexate; CS, corticosteroids; LEF, leflunomide; ETN, ertanercept; IFX: infliximab; AZ, azathioprine; TAC, tacrolimus; MMF, mycophenolate mofetil; CNS, central nervous system. †Magnetic resonance imaging consistent with cerebral vasculitis; might have been caused by coexistent *Aspergillus* infection.

**Table 2:** Diagnostic test results, management, and outcome for persons with *Anncaliia algerae* myositis*

Variable†	Case-patient A	Case-patient B	Case-patient 1 (*3*)	Case-patient 2 (*5*)	Case-patient 3 (*8*)
Hemoglobin, g/L (130–180)‡	95	122	ND	100	96
Lymphocytes ×10^9^/L (1.5–4.0)	0.3	0.4	ND	0.2	0.1
CK U/L, peak (<200)	2,028	6,630	6,337	685	441
ALT, U/L (<45)	154	93	ND	66	50
AST, U/L (<45)	320	210	ND	129	70
ESR, mm/hr (0–14)	85	26	ND	38	30
CRP, mg/L (<3)	152	134	ND	16	41
Serum albumin, lowest, g/L (33–48)	21	19	ND	19	14
Serum creatinine, µmol/L (60–100)§	44	202	ND	81	216
Urinary protein, g/24 h (<0.1)¶	0.56	1.8	ND	NT	1.53
Fecal stain#	No microsporidia	NT	ND	No microsporidia	No microsporidia
Neurophysiology/EMG	Myopathy; axonal neuropathy	Myopathy; axonal neuropathy	ND	Myopathy; axonal neuropathy	Myopathy; axonal neuropathy
Negative biopsy/fluid sites	CSF	Esophagus, stomach, duodenum	Tracheal aspirate	Bone marrow, lung, rectum, BAL, CSF	NT
Positive biopsy sites	Vastus lateralis	Vastus lateralis	Quadriceps femoris**	Deltoid, tongue	Deltoid
*A. algerae* sequence	Yes	Yes	Yes	Yes	Yes
Immunosuppression reduced	Yes	Yes	Yes	Yes	Yes
Albendazole	Yes	No	Yes	No	Yes
Outcome	Survived >18 mo	Died, aspiration pneumonia	Died, stroke	Died, palliated	Died, aspiration pneumonia

*ND, not described in publication; CK, creatinine kinase; ALT, alanine transaminase; AST, aspartate transaminase; ESR, erythrocyte sedimentation rate; CRP, C-reactive protein; EMG, electromyography; NT, not tested; CSF, cerebrospinal fluid; BAL, bronchoalveolar lavage. †Biochemical and hematologic values are from the most recent visit to hospital, unless otherwise indicated. Reference values are in parentheses. ‡Anemia was normocytic. §Case-patient A: serum creatinine was higher (150 μmol/L) at the first admission; case-patient B: baseline creatinine was ≈160μμmol/L; case-patient 3: preexisting renal impairment, with transplant. ¶Case-patient A: urinary myoglobin was negative; case-patient B urinary albumin:creatinine ratio 8 mo previously was normal; case-patient 3: urinary protein 2 mo previously was 0.68 g/24 h. #Modified trichrome stain on concentrated feces. **Component of quadriceps femoris not specified in publication.

## Case Reports

We have designated persons with the cases of *A. algerae* myositis described here as case-patients A and B and those with cases reported in 2004, 2012, and 2013 as case-patients 1, 2, and 3, respectively (*3*,*5*,*8*). We reviewed the medical records of case-patients 2 and 3 to obtain additional information.

### Case-patient A

In 2011, a 67-year-old man from coastal New South Wales, Australia, sought care at hospital for an 8-week history of watery diarrhea; weight loss; and increasing arthralgias, fatigue, lethargy, and generalized myalgias. He had rheumatoid factor–positive rheumatoid arthritis that was diagnosed when he was in his early twenties, with fluctuating joint disease, but no extra-articular involvement. Therapy preceding the illness included long-term methotrexate (20 mg weekly); leflunomide (20 mg/d), which was stopped 1 week before he sought care at a hospital; prednisone (5 mg/d); etanercept (antitumor necrosis factor α therapy) for 5 months; and nonsteroidal anti-inflammatory drugs (NSAIDs). Serum creatinine and liver function tests were moderately elevated. The methotrexate, etanercept, and NSAIDs were stopped; he responded to intravenous rehydration; his serum creatinine normalized; and he was discharged after 3 days.

The man’s symptoms progressed, dysphagia developed, and he was readmitted to the hospital 2 weeks later. He had generalized weakness in the upper and lower limbs of Medical Research Council grade 3+ to 4, distal greater than proximal, absent or reduced reflexes, and no clinical sensory abnormalities. Mild peripheral edema was present. He had mild glossitis but no mouth ulcers. He was noted to be at significant risk for aspiration because of bulbar muscle weakness and was not permitted to eat or drink by mouth; nasogastric feeding was begun. Prednisone was increased to 25 mg/d. His condition deteriorated despite 5 days of intravenous immunoglobulin for possible Guillain-Barré syndrome, and he was transferred to a Sydney tertiary referral hospital.

No cause for the diarrhea was found on fecal bacterial culture; examination for ova, cysts, and parasites; and modified trichrome staining for microsporidia (Table 2). A pulmonary infiltrate was present on computed tomographic (CT) scan. Results of magnetic resonance imaging of the brain and spinal cord were normal. Neurophysiologic studies showed reduced motor and sensory amplitudes and F wave persistence; electromyography demonstrated marked fibrillations and positive sharp waves, with only a mildly reduced pattern, suggesting mixed myositis and neuropathy.

**Figure 1:**
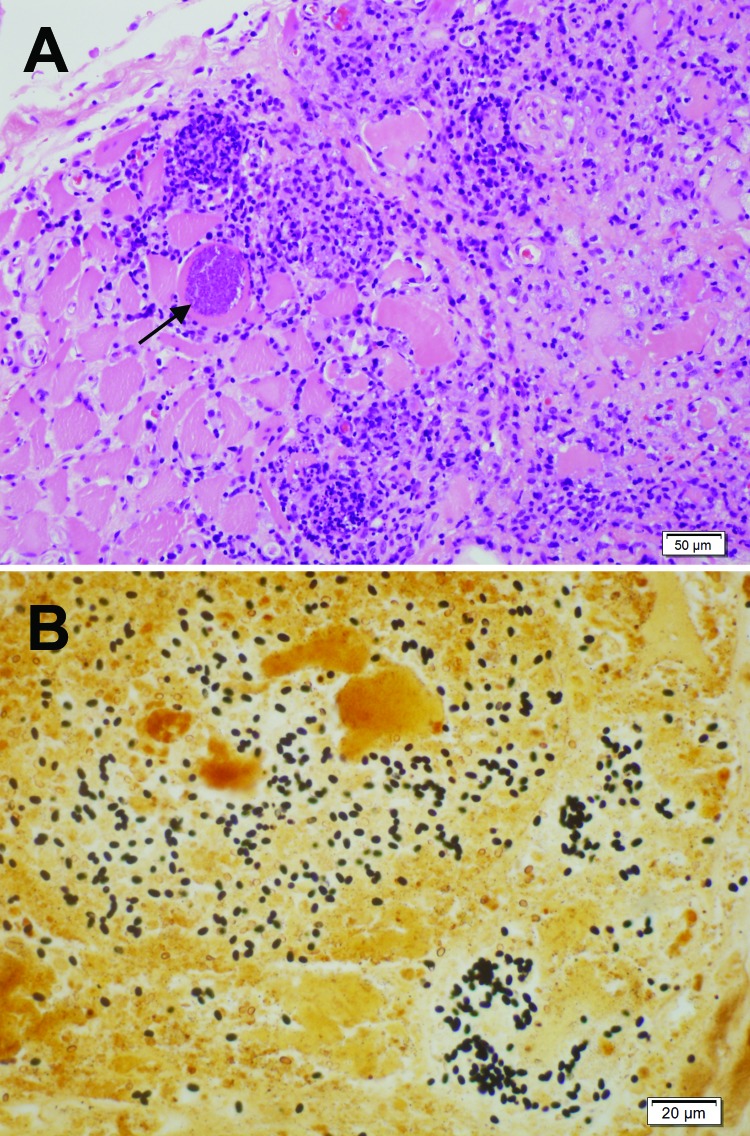
Light micrographs of muscle biopsy tissue from a 67-year-old man (case-patient A), New South Wales, Australia, showing microsporidial myositis caused by *Anncaliia algerae*. A) Necrotizing myositis with prominent inflammation and spores within the necrotic cytoplasm of a myocyte (arrow). Hematoxylin and eosin stain. Scale bar indicates 50 μm. B) Numerous dark brown to black, 3- to 4-μm ovoid spores in necrotic myocytes. Warthin-Starry stain. Scale bar indicates 20 μm.

**Figure 2:**
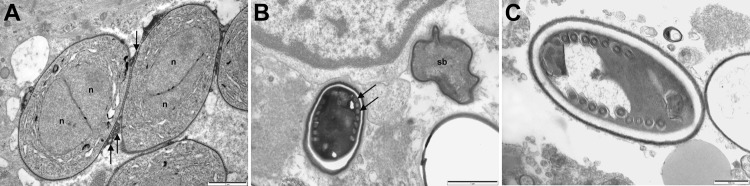
Electron micrographs of muscle biopsy tissue from a 66-year-old man (case-patient B) (A) and a 67-year-old man (case-patient A) (B,C) showing *Anncaliia algerae.* A) Early proliferative stage meronts with diplokaryotic nuclei (n) and vesiculotubular appendages (arrows) attached to the plasmalemma. Scale bar indicates 1 μm. B) Degenerate crenated sporoblast (sb) and a mature spore with visible coils of the polar tubule (arrows). Scale bar indicates 1 μm. C) Mature spore with 9 polar tubule coils in a single row, pale endospore, and a dense exospore. Scale bar indicates 500 nm.

An immediate vastus lateralis muscle biopsy was performed. Light microscopy demonstrated a necrotizing myositis with numerous ovoid spores (Figure 1). Electron microscopy confirmed the diagnosis of microsporidial myositis with features characteristic of *A. algerae* (Figure 2). DNA was extracted from the muscle biopsy specimen, and part of the small subunit ribosomal RNA gene was amplified by PCR in accordance with a published method (*5*). Sequence analysis was consistent with *A. algerae*.

Prednisone was reduced to 5 mg, and leflunomide was washed out with cholestyramine. Albendazole (400 mg 2×/d), sulfadiazine (1 g 4×/d) and pyrimethamine (50 mg/d) were begun. Fevers developed, and the patient’s condition continued to deteriorate for a week, reached a nadir, and progressively improved, despite cessation of albendazole after 3 weeks because of vomiting and abnormal liver function test results. Creatine kinase (CK) normalized at 12 months after hospital admission. At 18 months follow-up, there was no indication of infection, and the patient had regained full strength. His rheumatoid arthritis was controlled with NSAIDs, prednisone (5 mg/d), and injectable gold. Follow-up neurophysiology showed full recovery of nerve conduction velocities and amplitudes.

### Case-patient B

In 2006, a 66-year-old man from suburban Sydney, New South Wales, sought care at hospital for a 5-week history of progressive generalized muscle pain, lethargy, weight loss, poor appetite, low mood, difficulty sleeping, fevers, pain in the mouth, difficulty swallowing, and discomfort on opening the mouth. He had rheumatoid factor–positive rheumatoid arthritis diagnosed 2 years previously, with no extra-articular manifestations. He was treated with methotrexate for 22 months, with the dose gradually increased to 25 mg per week. This drug was stopped 2 weeks before hospital admission because of concerns about toxicity. Prednisone was begun 4 weeks before hospital admission (maximum dose 50 mg/d for 1 week).

The patient had fevers (maximum temperature 39.5°C); tachycardia; oropharyngeal mucositis; glossitis; macroglossia; a tongue ulcer; decreased tongue movements; trismus; peripheral edema; tenderness on palpation of the limbs; mild bilateral facial weakness; and upper and lower limb weakness, marked in the hands. Pain limited grading of the weakness; however, mobilization was possible. Sensation was normal, and reflexes were present.

Investigation results included methotrexate level <0.1 μmol/L, IgG (subtype 1) 1.65 g/L (reference 4.9–11.4) and (subtype 2) 0.78 g/L (reference 1.50–6.4), and troponin I 0.7 μg/L (reference <0.1) (Table 2). The urine contained no cellular casts. Transthoracic echocardiography showed normal left ventricular function. Noncontrast CT scan of the brain showed no abnormalities. Neurophysiologic studies revealed a mild axonal type neuropathy, and electromyography results were consistent with an active myopathic process in multiple muscles.

Broad-spectrum antimicrobial drug therapy, acyclovir, and intravenous immunoglobulin were given. The corticosteroid dose was reduced with a weaning regimen. Vastus lateralis muscle biopsies were performed ≈2 weeks into hospitalization. Histopathologic examination demonstrated large numbers of round, predominantly extracellular, 3- to 4-μm organisms. Specimens from esophageal, gastric, and duodenal biopsies did not contain organisms. Liposomal amphotericin B was begun to treat a presumed fungal infection.

The patient remained febrile, and his condition worsened. Nasogastric feeding was begun because of bulbar muscle weakness. Several other complications developed, including atrial fibrillation, delirium, and acute renal failure requiring dialysis. He died 4 weeks after admission after a nasopharyngeal bleed that led to aspiration pneumonia. Subsequent electron microscopy of the muscle biopsy demonstrated myositis caused by *A. algerae* (Figure 2). As with case-patient A, the partial sequence of the small subunit ribosomal RNA gene from the muscle biopsy was homologous with *A. algerae*.

## Discussion

In the cases reported here, *A. algerae* myositis caused fever, weight loss, fatigue, generalized muscle weakness and pain, dysphagia, glossitis, peripheral edema, and diarrhea (Table 1). The limb edema might have been multifactorial and associated with local inflammation of the muscle; hypoalbuminemia; and impaired renal function, including proteinuria (*19*). The urinary myoglobin was negative in case-patient A, and urinary sediment staining for microsporidia was either not performed or not documented in any of the case-patients. Three case-patients had diarrhea, but no microsporidia were observed in gut biopsy specimens or in stained fecal samples (*5*,*8*). Although oropharyngeal ulceration and mucositis in case-patients B and 3 might have been caused by medication, it also might represent *A. algerae* infection (*4*,*8*).

The cardiac and central nervous system disorders may be associated with the *A. algerae* infection, related to co-morbidities or nonspecific features of severe illness. Case-patient B had delirium and atrial fibrillation with increased troponin I. Case-patient 2 had delirium, seizures, and an increased troponin I, 1.32 μg/L (reference <0.01). Seizures were associated with *Trachipleistophora anthropophthera* dissemination to the brain (*16**,**17*). However, the signs of cortical vasculitis and ischaemia on magnetic resonance imaging of the brain in case-patient 2 may have been due to angio-invasive fungal infection, considering the concurrent *Aspergillus* lung abscess. Case-patient 1, who had type 2 diabetes mellitus, died of a cerebrovascular infarction (*3*).

Laboratory indicators of muscle damage and inflammation included elevated CK, aspartate aminotransferase, alanine aminotransferase, erythrocyte sedimentation rate, and C-reactive protein and decreased serum albumin (*20*) (Table 2). Peak CK levels were lower in the transplant patients. Lymphopenia and decreased immunoglobulin levels might have been related to preadmission medications, including corticosteroid therapy (*21*). Although the underlying causes are yet to be determined, anemia and proteinuria were common features. The resolution of the neuropathy on nerve conduction studies in case-patient A might indicate recovery from peripheral nerve infection.

After fixation in formalin and staining, biopsy specimens can be examined under light microscopy (*3*,*5*). Muscle biopsies show necrotic myocytes containing spores, which stain poorly with hematoxylin and eosin (Figure 1, panel A) (*5*). Silver stains, particularly the Warthin-Starry, clearly demonstrate 3- to 4-μm ovoid spores (Figure 1, panel B) (*5*,*22*). In case-patient 1, muscle was immunofluorescently stained by using antibodies to *A. algerae* (*3*). The differential diagnoses include the yeast forms of fungi and leishmania amastigotes.

Transmission electron microscopy of biopsies can confirm a diagnosis of microsporidial myositis and differentiate *Anncaliia* spp. from *Trachipleistophora hominis* (*3*,*5*,*23*). Although electron microscopy is highly specific for microsporidiosis, the number of organisms within a biopsy specimen limits sensitivity. Features that define *Anncaliia* spp. include binary fission; diplokaryotic nuclei; lack of a parasitophorous vacuole, with the thickened plasmalemma of meronts and sporonts in direct contact with host cytoplasm; and vesiculotubular appendages projecting from the plasmalemma (Figure 2, panel A) (*5*,*23*). Degenerate sporoblasts and spores appear crenated (Figure 2, panel B). Mature spores have a dense exospore coat, a pale endospore, and single rows of 8–11 polar tubule coils (Figure 2, panels B, C). This number of polar tubule coils and the absence of deep protoplasmic extensions are features of *A. algerae*; however, species identification also relies on nucleic acid sequence homology (*5*,*23*). DNA extracted from clinical specimens can be amplified by PCR (*3*,*5*). The primers target areas of the large and small subunit ribosomal RNA genes and sequence analysis enables identification of the organism.

For case-patient A, who survived, a muscle biopsy was performed as soon as it became evident that empiric corticosteroid and intravenous immunoglobulin therapy was not effective. The corticosteroid dose was minimized; etanercept, methotrexate, and leflunomide had been stopped; and leflunomide was washed out with cholestyramine (*24*). Measures were taken to avoid aspiration pneumonia. On histopathology, microsporidiosis was considered, and a regimen with albendazole was begun. For case-patients B and 2, in whom microsporidiosis was diagnosed postmortem, antifungal agents were used. In vitro studies indicated that amphotericin B had no activity against the tested microsporidia (*25*).

Albendazole targets microtubules and prevents formation of intranuclear spindles in microsporidia (*25*). Microsporidial myositis caused by species other than *A. algerae* has been responsive to albendazole alone or in combination with other agents (*9*,*12*,*18*). Although albendazole has not been tested for activity against *A. algerae*, the survival of case-patient A suggests clinical effectiveness. Side effects include headache, gastrointestinal upset, and elevated levels of transaminases (*25*).

In case-patient A and a case-patient with *Trachipleistophora* myositis, the combination of albendazole, sulfadiazine, and pyrimethamine was successful (*12*). However, the contribution of sulfadiazine and pyrimethamine to the regimen is unclear because in vitro testing has indicated these agents are ineffective against the microsporidian *Encephalitozoon cuniculi* (*26*). Metronidazole, itraconazole, atovaquone, and clindamycin were among the medications used for case-patient 1 (*3*). The activity of these agents against microsporidia varies (*25*,*27*). Fumagillin and its analogs have been used topically to treat corneal infections and orally for gastrointestinal microsporidiosis (*27*), but because of possible side effects, they have not been used to treat microsporidial myositis (*27*).

Like other microsporidia, *A. algerae* depends on host cells to obtain metabolites (*28*). Genes that code for a diversity of kinases and transport proteins are regarded as an adaptation to the broad range of insect hosts (*7*,*28*). *A. algerae* was previously investigated as a biologic control agent for mosquitoes because heavily infected larvae rarely survive to adulthood (*7*). Other than in humans, no naturally occurring infections have been identified in mammals.

Mosquitoes become infected during their aquatic immature stages, either through exposure to spores in the water or by feeding on dead insect hosts (*7*,*29*). Infection occurs when the polar tubule is extruded from the spore and sporoplasm is introduced into the host cell through the tubule (*7*). In the initial proliferative phase, asexual reproduction occurs (*7*,*29*). Sporoloblasts then mature into spores (*7*,*29*). Although spores survive outside the host, they become nonviable when dried at room temperature for 5 days (*30*,*31*). Information is limited about the life cycle of *A. algerae* in Australia. *Culex sitiens* Wiedemann, an abundant coastal mosquito that usually bites birds, is currently the only documented insect host (*7*,*32*).

The exact mode of transmission of *A. algerae* to humans is yet to be established. Contact with water that contains spores generated in insect hosts is thought to be the most likely mechanism (*6*,*33*). Mechanical inoculation of spores by crushing a feeding mosquito into a bite wound has also been proposed as means a transmission (*3*,*7*). However, spores from mosquito tissue have substantially lower germination rates in human plasma or serum than those first exposed to water (*34*). Direct inoculation from a mosquito bite also is considered less likely because the saliva of laboratory-infected mosquitoes did not contain *A. algerae* (*30*). The Australian case-patients lived on the eastern seaboard, within 500 km of each other, in a warm temperate environment. They were exposed to mosquito habitat through proximity to bodies of water on golf courses or coastal woodland.

All the 5 case-patients reviewed were taking immunosuppressant therapy, and 2 had coexistent diabetes mellitus. Before infection, methotrexate, leflunomide, corticosteroids, and antitumor necrosis factor α therapy were used as treatments in the case-patients with rheumatoid arthritis. Epidemiologic studies have suggested that methotrexate and leflunomide are not associated with a substantial increase in serious infection, although this lack of association does not exclude a contribution with combination immunosuppression (*35*–*37*). Antitumor necrosis factor α therapy and corticosteroids are associated with increased risk (*35*,*38*,*39*). The severity of rheumatoid arthritis is also a risk factor for serious infection (*35*). However, before the addition of corticosteroids 4 weeks before hospital admission, methotrexate was the only immunosuppressive agent used by case-patient B, and he had no erosive or extraarticular disease.

Case-patients 2 and 3 were solid-organ transplant recipients (*5*). The immunosuppressive agents used in the months preceding the development of *A. algerae* myositis were mycophenolate mofetil, tacrolimus, azathioprine, and corticosteroids. Systemic infections caused by *Encephalitozoon* spp. in solid-organ transplants have been associated with these therapies (*40*). Studies with *E. cuniculi* have indicated the importance of cytotoxic CD8+ T-cell immunity for protection against severe and persistent infection (*1*).

*A. algerae* myositis is uncommon infection and has a high case-fatality rate. Whether it has emerged because previous cases have gone unrecognized or because epidemiologic factors have changed is unclear. *A. algerae* transmission occurs possibly through contact with water that contains spores amplified by insect hosts. The signs and symptoms of *A. algerae* infection primarily indicate myositis. Whether other organs and tissues may be infected has yet to be confirmed. In case-patient A, who survived, microsporidiosis was diagnosed on biopsy, and clinical management was based on the reduction of immunosuppression, measures to prevent complications, and a treatment regimen based on albendazole. Genomic analysis for drug targets and susceptibility testing of *A. algerae* may lead to increased therapeutic options. *A. algerae* myositis is an additional differential diagnosis to be considered in immunocompromised persons.

## References

[1] Ghosh K, Weiss LM. T cell response and persistence of the microsporidia. FEMS Microbiol Rev. 2012;36:748–60. 10.1111/j.1574-6976.2011.00318.x 22126330

[2] Visvesvara GS, Belloso M, Moura H, Da Silva AJ, Moura IN, Leitch GJ, Isolation of *Nosema algerae* from the cornea of an immunocompetent patient. J Eukaryot Microbiol. 1999;46:10S . 10519226

[3] Coyle CM, Weiss LM, Rhodes LV III, Cali A, Takvorian PM, Brown DF, Fatal myositis due to the microsporidian *Brachiola algerae*, a mosquito pathogen. N Engl J Med. 2004;351:42–7. 10.1056/NEJMoa032655 15229306 PMC3109631

[4] Cali A, Neafie R, Weiss LM, Ghosh K, Vergara RB, Gupta R, Human vocal cord infection with the microsporidium *Anncaliia algerae.* J Eukaryot Microbiol. 2010;57:562–7. 10.1111/j.1550-7408.2010.00510.x 20958855 PMC3109663

[5] Field AS, Paik JY, Stark D, Qiu MR, Morey A, Plit ML, Myositis due to the microsporidian *Anncaliia* (*Brachiola*) algerae in a lung transplant recipient. Transpl Infect Dis. 2012;14:169–76. 10.1111/j.1399-3062.2012.00724.x 22385431

[6] Visvesvara GS, Moura H, Leitch GJ, Schwartz DA, Xiao LX. Public health importance of *Brachiola algerae* (*Microsporidia*)—an emerging pathogen of humans. Folia Parasitol (Praha). 2005;52:83–94 . 16004367 10.14411/fp.2005.011

[7] Andreadis TG. Microsporidian parasites of mosquitoes. J Am Mosq Control Assoc. 2007;23(Suppl):3–29 and. 10.2987/8756-971X(2007)23[3:MPOM]2.0.CO;2 17853594

[8] Chacko B, Trevillian P. Microsporidial myositis in a kidney transplant recipient [abstract 82]. Program and abstracts of Annual Scientific Meeting Transplantation Society of Australia and New Zealand. Canberra (ACT): Transplantation Society of Australia and New Zealand; 2013. p. 96.

[9] Cali A, Takvorian PM, Lewin S, Rendel M, Sian CS, Wittner M, *Brachiola vesicularum*, n. g., n. sp., a new microsporidium associated with AIDS and myositis. J Eukaryot Microbiol. 1998;45:240–51. 10.1111/j.1550-7408.1998.tb04532.x 9627985

[10] Margileth AM, Strano AJ, Chandra R, Neafie R, Blum M, McCully RM. Disseminated nosematosis in an immunologically compromised infant. Arch Pathol. 1973;95:145–50 . 4686506

[11] Choudhary MM, Metcalfe MG, Arrambide K, Bern C, Visvesvara GS, Pieniazek NJ, *Tubulinosema* sp. microsporidian myositis in immunosuppressed patient. Emerg Infect Dis. 2011;17:1727–30. 10.3201/eid1709.101926 21888805 PMC3322067

[12] Field AS, Marriott DJ, Milliken ST, Brew BJ, Canning EU, Kench JG, Myositis associated with a newly described microsporidian, *Trachipleistophora hominis*, in a patient with AIDS. J Clin Microbiol. 1996;34:2803–11 . 8897186 10.1128/jcm.34.11.2803-2811.1996 PMC229407

[13] Curry A, Beeching NJ, Gilbert JD, Scott G, Rowland PL, Currie BJ. *Trachipleistophora* hominis infection in the myocardium and skeletal muscle of a patient with AIDS. J Infect. 2005;51:e139–44. 10.1016/j.jinf.2004.11.006 16230193

[14] Chupp GL, Alroy J, Adelman LS, Breen JC, Skolnik PR. Myositis due to *Pleistophora* (*Microsporidia*) in a patient with AIDS. Clin Infect Dis. 1993;16:15–21. 10.1093/clinids/16.1.15 8448294

[15] Cali A, Takvorian PM. Ultrastructure and development of *Pleistophora ronneafiei* n. sp., a microsporidium (Protista) in the skeletal muscle of an immune-compromised individual. J Eukaryot Microbiol. 2003;50:77–85. 10.1111/j.1550-7408.2003.tb00237.x 12744518

[16] Vávra J, Yachnis AT, Shadduck JA, Orenstein JM. Microsporidia of the genus *Trachipleistophora*—causative agents of human microsporidiosis: description of Trachipleistophora anthropophthera n. sp. (Protozoa: Microsporidia). J Eukaryot Microbiol. 1998;45:273–83. 10.1111/j.1550-7408.1998.tb04536.x 9627987

[17] Yachnis AT, Berg J, Martinez-Salazar A, Bender BS, Diaz L, Rojiani AM, Disseminated microsporidiosis especially infecting the brain, heart, and kidneys. Report of a newly recognized pansporoblastic species in two symptomatic AIDS patients. Am J Clin Pathol. 1996;106:535–43 . 8853044 10.1093/ajcp/106.4.535

[18] Suankratay C, Thiansukhon E, Nilaratanakul V, Putaporntip C, Jongwutiwes S. Disseminated infection caused by novel species of *Microsporidium*, Thailand. Emerg Infect Dis. 2012;18:302–4. 10.3201/eid1802.111319 22305387 PMC3310463

[19] Lee KH, Lim SR, Kim YJ, Lee KJ, Myung DS, Jeong HC, Acute dermatomyositis associated with generalized subcutaneous edema. Rheumatol Int. 2008;28:797–800. 10.1007/s00296-008-0520-0 18193426

[20] Volochayev R, Csako G, Wesley R, Rider LG, Miller FW. Laboratory test abnormalities are common in polymyositis and dermatomyositis and differ among clinical and demographic groups. Open Rheumatol J. 2012;6:54–63. 10.2174/1874312901206010054 PMC3377888 22723809

[21] Klaustermeyer WB, Gianos ME, Kurohara ML, Dao HT, Heiner DC. IgG subclass deficiency associated with corticosteroids in obstructive lung disease. Chest. 1992;102:1137–42. 10.1378/chest.102.4.1137 1343817

[22] Field AS, Hing MC, Milliken ST, Marriott DJ. Microsporidia in the small intestine of HIV-infected patients. A new diagnostic technique and a new species. Med J Aust. 1993;158:390–4 . 7683076

[23] Franzen C, Nassonova ES, Scholmerich J, Issi IV. Transfer of the members of the genus *Brachiola* (microsporidia) to the genus *Anncaliia* based on ultrastructural and molecular data. J Eukaryot Microbiol. 2006;53:26–35. 10.1111/j.1550-7408.2005.00066.x 16441582

[24] Jenks KA, Stamp LK, O'Donnell JL, Savage RL, Chapman PT. Leflunomide-associated infections in rheumatoid arthritis. J Rheumatol. 2007;34:2201–3 . 17937473

[25] Costa SF, Weiss LM. Drug treatment of microsporidiosis. Drug Resist Updat. 2000;3:384–99. 10.1054/drup.2000.0174 11498405

[26] Beauvais B, Sarfati C, Challier S, Derouin F. In vitro model to assess effect of antimicrobial agents on *Encephalitozoon cuniculi.* Antimicrob Agents Chemother. 1994;38:2440–8 and. 10.1128/AAC.38.10.2440 7840584 PMC284758

[27] Didier ES, Maddry JA, Brindley PJ, Stovall ME, Didier PJ. Therapeutic strategies for human microsporidia infections. Expert Rev Anti Infect Ther. 2005;3:419–34. 10.1586/14787210.3.3.419 15954858

[28] Peyretaillade E, Parisot N, Polonais V, Terrat S, Denonfoux J, Dugat-Bony E, Annotation of microsporidian genomes using transcriptional signals. Nat Commun. 2012;3:1137. 10.1038/ncomms2156 23072807

[29] Vavra J, Undeen AH. *Nosema algerae* n. sp. (*Cnidospora, Microsporida*) a pathogen in a laboratory colony of *Anopheles stephensi* Liston (Diptera, Culicidae). J Protozool. 1970;17:240–9. 10.1111/j.1550-7408.1970.tb02365.x 4915459

[30] Trammer T, Dombrowski F, Doehring M, Maier WA, Seitz HM. Opportunistic properties of *Nosema algerae* (*Microspora*), a mosquito parasite, in immunocompromised mice. J Eukaryot Microbiol. 1997;44:258–62. 10.1111/j.1550-7408.1997.tb05709.x 9183715

[31] Alger NE, Undeen AH. The control of a microsporidian, *Nosema* sp., in an anopheline colony by an egg-rinsing technique. J Invertebr Pathol. 1970;15:321–7. 10.1016/0022-2011(70)90177-1 4987976

[32] Webb CE, Russell RC. Towards management of mosquitoes at Homebush Bay, Sydney, Australia. I. Seasonal activity and relative abundance of adults of *Aedes vigilax, Culex sitiens*, and other salt-marsh species, 1993–94 through 1997–98. J Am Mosq Control Assoc. 1999;15:242–9 . 10412120

[33] Cali A, Weiss LM, Takvorian PM. A review of the development of two types of human skeletal muscle infections from microsporidia associated with pathology in invertebrates and cold-blooded vertebrates. Folia Parasitol (Praha). 2005;52:51–61 . 16004364 10.14411/fp.2005.007 PMC3109649

[34] Undeen AH, Alger NE. Nosema algerae: infection of the white mouse by a mosquito parasite. Exp Parasitol. 1976;40:86–8. 10.1016/0014-4894(76)90068-0 950003

[35] Doran MF, Crowson CS, Pond GR, O'Fallon WM, Gabriel SE. Predictors of infection in rheumatoid arthritis. Arthritis Rheum. 2002;46:2294–300. 10.1002/art.10529 12355476

[36] Salliot C, van der Heijde D. Long-term safety of methotrexate monotherapy in patients with rheumatoid arthritis: a systematic literature research. Ann Rheum Dis. 2009;68:1100–4. 10.1136/ard.2008.093690 19060002 PMC2689525

[37] Lacaille D, Guh DP, Abrahamowicz M, Anis AH, Esdaile JM. Use of nonbiologic disease-modifying antirheumatic drugs and risk of infection in patients with rheumatoid arthritis. Arthritis Rheum. 2008;59:1074–81 . 10.1002/art.23913 18668604

[38] Dixon WG, Abrahamowicz M, Beauchamp ME, Ray DW, Bernatsky S, Suissa S, Immediate and delayed impact of oral glucocorticoid therapy on risk of serious infection in older patients with rheumatoid arthritis: a nested case-control analysis. Ann Rheum Dis. 2012;71:1128–33. 10.1136/annrheumdis-2011-200702 22241902 PMC3375584

[39] Galloway JB, Hyrich KL, Mercer LK, Dixon WG, Fu B, Ustianowski AP, Anti-TNF therapy is associated with an increased risk of serious infections in patients with rheumatoid arthritis especially in the first 6 months of treatment: updated results from the British Society for Rheumatology Biologics Register with special emphasis on risks in the elderly. Rheumatology (Oxford). 2011;50:124–31. 10.1093/rheumatology/keq242 20675706 PMC3105607

[40] Lanternier F, Boutboul D, Menotti J, Chandesris MO, Sarfati C, Mamzer Bruneel MF, Microsporidiosis in solid organ transplant recipients: two *Enterocytozoon bieneusi* cases and review. Transpl Infect Dis. 2009;11:83–8. 10.1111/j.1399-3062.2008.00347.x 18803616

